# A Comprehensive Intervention for Promoting Successful Aging Amongst Older People With Diabetes With Below-Normal Cognitive Function—A Feasibility Study

**DOI:** 10.3389/fendo.2020.00348

**Published:** 2020-06-02

**Authors:** Rachel Natovich, Noa Gayus, Michal Azmon, Hila Michal, Omri Gury Twito, Tomer Yair, Svetlana Raudoi, Ori Kapra, Tali Cukierman-Yaffe

**Affiliations:** ^1^Endocrinology Division, The Center for Successful Aging With Diabetes, Sheba Medical Center, Gertner Institute, Ramat Gan, Israel; ^2^Sheba Medical Center, The Rehabilitation Hospital, Ramat Gan, Israel; ^3^The Physiotherapy Department, Faculty of Health Sciences, Ariel University, Ariel, Israel; ^4^The Epidemiology Department, Sackler School of Medicine, The Herczeg Institute on Aging, Tel-Aviv University, Ramat Gan, Israel

**Keywords:** cognition, diabetes, A1C, aging, intervention

## Abstract

**Background:** Older people with diabetes have an increased risk for disability and cognitive dysfunction, which may impede self-care capacity. These are not evaluated routinely in current health systems. In the Center for Successful Aging with Diabetes, patients over the age of 60 undergo multi-disciplinary evaluation days and are provided with an integrated (cognitive, physical, nutritional, and medical) treatment plan. Among individuals with below-normal cognitive function, self-adherence to these recommendations poses a challenge. Thus, the aim of this study was to test the feasibility of a multidisciplinary intervention amongst older people with diabetes with below-normal cognitive function and sub-optimal glucose control.

**Methods:** Patients with a MoCA score under 26 and A1C >= 7.5% participated in a two-arm intervention: (A) a medical intervention: monthly meetings with a diabetes nurse-educator, supervised by a diabetes specialist and study psychologist during which changes in their pharmacological regimen of glucose, blood pressure, and lipid control were made and (B) a cognitive/physical rehabilitation intervention. This arm consisted of (1) an intensive phase-group meetings which included computerized cognitive training, aerobic, balance, and strength exercise, and group discussions and (2) a monthly consolidation phase. Outcomes included change in A1C, change in strength, balance, and aerobic exercise capacity as well as change in quality of life.

**Results:** After 12 months there was a 0.7% reduction in A1C. After 3 months there was a statistically significant improvement in physical indices, including aerobic capacity (6-min walk), balance (FSST) and indices assessing the risk of fall (10-meter walk, time up and go). There was no additional improvement in physical indices between the 3 and 12 month visits. For some of the physical measures, the improvement observed after 3 months persisted partially to the 12-month visit.

**Conclusions:** This feasibility study provides preliminary data that support the efficacy of the complex interventions described. The findings suggest that this older population would require an ongoing “intensive phase” intervention. Larger prospective randomized trials are needed.

## Introduction

The prevalence of diabetes increases with age. In the US it has been reported that ~25–30% of the population over 65 have diabetes (1). In Israel, it has been reported that ~23% of those over the age of 65 have a diagnosis of diabetes (2). Diabetes is well-established as a risk factor for eye, kidney, and neurological diseases as well as for cardiovascular morbidity and mortality. Data from the last several years has shown that it is also a risk factor for cognitive dysfunction (3), dementia (4–6), and disability (7). These have been shown to impede patients' self-care management capacities (1, 8, 9). Thus, many current guidelines recommend screening and survillence of cognitive status so that treatment plan may be appropriatly tailored (10–12).

Type 2 diabetes is a disease that requires complex self-care management capacities, including a variety of health-related behaviors such as diet, physical activity, adherence to medication, medical surveillance, and self- inspection. Self-care management in diabetes has been shown to be important in all age groups, with positive effects on glucose control and in the prevention of the long-term negative consequences of diabetes (10). There is a large body of data supporting the multidisciplinary treatment team approach (MDT) as the evidence-based disease management strategy for promoting self-care management (11, 12). Adherence to MDT requires that the person with diabetes learn and understand new information and treatment procedures provided by various health professionals (physician, nurse-educator, dietitian, etc.), manage his disease condition (self-inspection, setting up medical appointments, etc.) and make changes in life habits. These treatment demands represent also an increase in cognitive demands needed for the optimal implementation of medical recommendations. Cognitively, the person with diabetes is required to learn and understand new information, remember it, plan and initiate self-treatment, apply behavioral changes using psychomotor capacities and perseverance abilities, while at the same time controlling and repressing impulses.

Many diabetes clinics provide patients with routine surveillance and multi-disciplinary treatment for the well-known complications of diabetes (for example eye and kidney disease) as well as glucose, blood pressure, and lipid control as standard of care in treatment of people with diabetes. However, these clinics cater to all age groups and disregard the special needs of older patients, including mobility, frailty, and cognitive and communication difficulties, which impact the potential utilization of health care services. At the center for successful aging with diabetes we aim to overcome these barriers by offering a novel comprehensive evaluation that incorporates cognitive, physical and emotional assessments that together enable the tailoring of a personalized treatment plan (13). Among individuals with below-normal cognitive function, self-adherence to these recommendations poses a challenge. Thus, the aim of this study was to test the feasibility of a group cognitively-oriented MDT program and assess its effect on glucose control, quality of life, and specific physical indices in older individuals with diabetes who have below-normal cognitive function and sub-optimal glucose control.

## Materials and Methods

This was a feasibility study. From the cohort of individuals who had undergone evaluation at the center for successful aging with diabetes (13) people with a MoCA score of below 26 and also an A1C of >= 7.5 were approached and asked to participate in the study.

**Figure 1:**
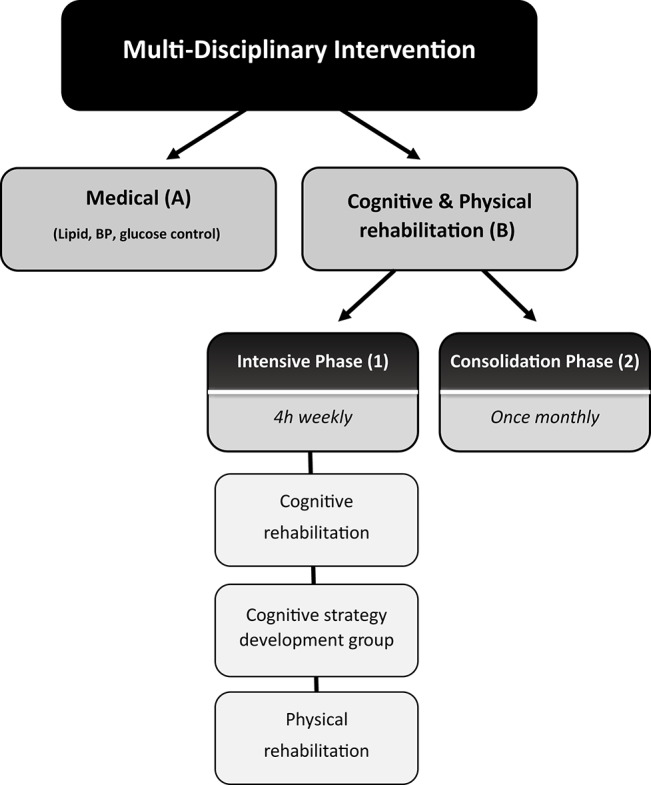
Schematic description of the complex multi-disciplinary intervention. The intervention consisted of a two-arm intervention: **(A)** a medical intervention: monthly meetings with a diabetes nurse-educator, supervised by a diabetes specialist and study psychologist during which changes in their pharmacological regimen of glucose, blood pressure, and lipid control were made, and **(B)** a cognitive/physical rehabilitation intervention. This arm consisted of (1) an intensive phase-group meetings which included computerized cognitive training, aerobic, balance, and strength exercise, and group discussions and (2) a monthly consolidation phase.

Individuals participated in a 2-arm intervention (Figure 1).

(A) A medical intervention involving monthly meeting with a diabetes nurse-educator supervised by a diabetes specialist and consulted by a study psychologist, in which changes in pharmacological regimen of glucose, blood pressure, and lipid control were made.

(B) A multi-disciplinary group intervention consisting of:

(1) An intensive phase. Weekly 4-h group meeting which included individually tailored computerized cognitive training sessions, aerobic, balance, strength exercise, and group discussion which were dedicated to cognitive rehabilitation strategies development and implementation with emphasis on disease management and physical activity as well as psycho-education on various disease management aspects (medical and nutritional). Computerized cognitive training was conducted using the Brain HQ cognitive training program. The program has 29 online exercises that work on many cognitive domains including attention, speed, memory, people skills, navigation, and intelligence. The participants got free access to BrainHQ and were asked to exercise at least three times a week. In the weekly meeting sessions, participants were provided with a tablet and each session was dedicated to a different cognitive domain. The importance of each cognitive domain in diabetes self-care performance was explained. Each participant was required to perform cognitive tasks in increasing level of complexity according his/her abilities. Neuropsychologists led the sessions.

Each week they received physical and cognitive tasks to conduct on a daily basis at home, and the performance of these tests was monitored during the weekly meeting.

(2) A consolidation phase monthly 2-h group discussions on challenges of implementation and coping strategies.

Both phases were conducted by a neuropsychologist and physiotherapist. Psycho-education on medical and nutritional aspects were conducted by a diabetes nurse educator, diabetes specialist and dietitian.

Outcomes included change in A1C (primary), change in aerobic, strength, and balance indices (including the 6-min walk (14–16), 10-meter walk (17, 18), Four Step Square Test-FSST (19), timed Up & Go (TUG) (20–23) 30-s chair stand (24) and grip strength using a Jammer dynamometer) (25) as well as change in quality of life [the WHO-5 well-being questionnaire (26), the single general self-rated health question (27)]. As follow-up was only 12 months a-priori we did not hypothesize that there would be a detectable difference in cognitive function and thus did not assess it at end of follow-up. A long-term follow-up study is planned, in which among others cognitive function will be assessed.

After 3 months, and again at the end of study A1C, physical indices and quality of life were assessed. Additionally participants filled out a self-administered questionnaire regarding their experience and were asked to grade from 1 to 10 their satisfaction with the program.

### Measurement

#### Timed Up and Go (20–23)

The objective of this test is to measure the ability of a person to: stand up, walk, turn around and sit down safely in a timely manner. The test examines most mobility skills. The participant is told to get up from a chair with handles, walks 3 meters, turns, walks back, and sit down again. The score is according to the length of time in seconds to complete the task. The score is categorized according to the risk for falls and independent walking. The following cut-offs are conventionally used: <14 s = independent mobility; 15–20 s = semi-independent mobility may have a some what increased risk for falls & needs further evaluation some may need a walking aid; 20–30 s = dependent mobility: need help walking, 50% with a cane, 40% walker, 10% supervision. Some will need help in transfers, and most will require help using the toilet. Many in this category won't go outside the home alone.

Data suggests that the timed “Up & Go” test is a reliable and valid test for quantifying functional mobility that may also be useful in following clinical change over time.

#### 6 min Walk (14–16)

The six-minute walk test (6 MWT) measures the distance an individual is able to walk over a total of 6 min on a hard, flat surface. The goal is for the individual to walk as far as possible in 6 min. The individual is allowed to self-pace and rest as needed as they traverse back and forth along a marked walkway. The six minute walk distance in healthy adults has been reported to range from 400 to 700 m. People with lower vs. higher scores on the 6-min walk are at higher risk for falls, disability, frailty, hospitalization, and death.

#### 10 Meter Walk (17, 18)

The test examines the pace and number of steps it takes a person to pass 10 meters. A route of 10 meters is marked by two lines and a chair is placed two meters past the runway end line. The subject starts the test two meters before the runway and goes 14 meters (two meters for acceleration at the beginning and two meters for deceleration at the end). The score achieved is determined by the time lapsed by the participant during walking along the middle 10 meters. Subject performs the test four times, the first two times are for practice: measurement occurs only during the third and fourths time. In addition to measuring the speed, the number of steps required to cross the short distance are also counted. Studies have a shown that better gait speed is associated with a lower risk for functional decline, hospitalization and mortality.

#### Four Square Step Test (FSST) (19)

The test evaluates dynamic balance in a high functional level and features walk forward backwards left and right above 2, 90 and 2.5 cm high long sticks that divide the floor into four squares. The participant is stands in square 1 facing no. 2 square. The goal is to walk as quickly as possible in all the squares in the following order: from 1 to- 2,3,4,1,4,3,2, and 1 without touching the sticks. The score is the time required to complete the entire route. A score of above 15 s has a high positive predictive value for a high risk for falls.

#### Grip Strength (25)

The maximum grip strength test is measured using the Jammer dynamometer. The score is the average in kilograms. This score is compared to the general population according to age and sex (25). Studies show that the grip decreases after midlife. Studies demonstrate a relationship between lower scores and a higher risk for falls, disability, health-related quality of life, longer hospitalization, and death.

#### The 30 s Sit to Stand Test (24)

This test examines the strength of the lower extremities. The participant is instructed to stand up for a full session as many times as he can, without the help or push of the hands (his hands crossed on his chest) for 30 s. The score is determined by the number of times the subject is able to achieve full compliance. The score on this test has been shown to have good discriminatory ability with respect to the risk for falls.

#### Definition of Other Variables

Medical variables were collected through interview, physical examination and collection of information from medical records. Neuropathy was defined as either bilateral reported neuropathy or bilateral reduced pain, touch vibration sensation on physical examination of lower limbs. Retinopathy was defined as evidence of diabetic retinopathy on eye examination. Diabetic Kidney Disease (DKD) was defined as either an elevated creatinine level or a microalbumin/creatinine ratio of above 30 mg/gram. Severe hypoglycemia was defined as a reported episode of hypoglycemia requiring the aid of another.

The study was approved by the Sheba medical center IRB committee and all participants signed an informed consent form.

#### Analysis

Baseline demographic, medical, and psycho-social characteristics collected during the evaluation processes were presented using mean (SD), N(%), medians, and Intra quartile Range (IQR). The difference in median scores between baseline measurement and after 3 months of the interventions were calculated, and statistical significance was tested using the Kruskal-Wallis/Mann-Whitney U test. The statistical significance of the trend between measurements (3, 12 months) and baseline parameters was calculated using a mixed model with repeated measures analysis with the Tukey-Kramer correction for multiple comparisons using Proc Mixed in SAS Version 9.1.3 (SAS Institute Inc. Cary, NC, USA). All *p*-values < 0.05 were considered significant.

## Results

Twenty-one individuals with type 2 diabetes with a MoCA score of below 26 and also an A1C of >=7.5 were included in the study. The demographic and medical characteristics of study participants is depicted in Table 1. As can be seen, mean age was 73, there were more men than women, and the cohort was relatively well-educated. Mean diabetes duration was 19 years with a mean A1C of 8.8%, 76% were insulin users, 43% had previous Ischemic Heart Disease (IHD), 24% previous CVD, and 14% reported a previous event of severe hypoglycemia requiring the aid of another.

**Table 1:** Demographic & medical characteristics.

**Variable**	**%(N); Mean(SD)**
Age	73 (4)
Female	38 (8)
Education	14 (4)
Smoking	14 (3)
Dyslipidemia	71 (15)
HTN	76 (16)
Diabetes duration(Y)	19 (9)
A1C(%)	8.8 (0.99)
Experienced severe hypoglycemia	14 (3)
Neuropathy	43 (9)
Retinopathy	5 (1)
Nephropathy	38 (8)
IHD	43 (9)
CVD	24 (5)
Insulin user	76 (16)
Statin use	71 (15)

Table 2 presents the baseline cognitive, psycho-social self-care characteristics of the cohort. As can be seen, depression symptom score was low (indicating lack of depressive symptoms), and cognitive scores were below the norm in many of the cognitive tests utilized. Reported self-care ability was high for adherence to medication but lower for adherence to diet, physical activity, and foot care.

**Table 2:** Baseline Cognitive, Psycho-Social, Self-care Characteristics.

**Variable**	**Mean (SD)**	**Median (IQR)**
PHQ-9	4.6 (3.9)	4 (2, 6)
MOCA	22.7 (2.8)	24 (20, 25)
DSST	8.1 (1.9)	8 (7, 9)
VF phonetic	−0.51 (1.3)	−0.5 (−1.3, 0.5)
VF semantic	−0.55 (1)	−0.54 (−1.1, 0.04)
Neurotrax global cognitive score	95.8 (5.7)	95 (92.4, 99)
Neurotrax memory score	97 (13.3)	97 (88.3, 107.2)
Neurotrax executive function score	93.3 (8.6)	96 (90.1, 101.9)
Neurotrax attention score	93.7 (11.9)	97.3 (83.5, 102.6)
Neurotrax motor skills	97.1 (6.5)	96.6 (92.1, 101.8)
SDSCA diet score	4.06 (1.05)	4.2 (3.43, 4.9)
SDSCA exercise score	1.17 (1.09)	1 (0, 2)
SDSCA blood glucose testing	5.1 (2.7)	7 (4.5, 7)
SDSCA adherence to medication	7 (0)	7 (0)
SDSCA foot care	3.9 (1.9)	4.2 (2.6, 5.4)

*Patient Health Questionnaire (PHQ-9) for depression symptom assessment (28); Montreal Cognitive Assessment (MoCA) (29); Digit Symbol Substitution Test (DSS) (30, 31); Verbal Fluency (VF) (32); Mindstreams (NeuroTrax) Cognitive Assessment (33); The summery of diabetes self-care activity (SDSCA) (34)*.

### Difference in Outcomes at the End of Intensive Phase (3-Months)

**Figure 2:**
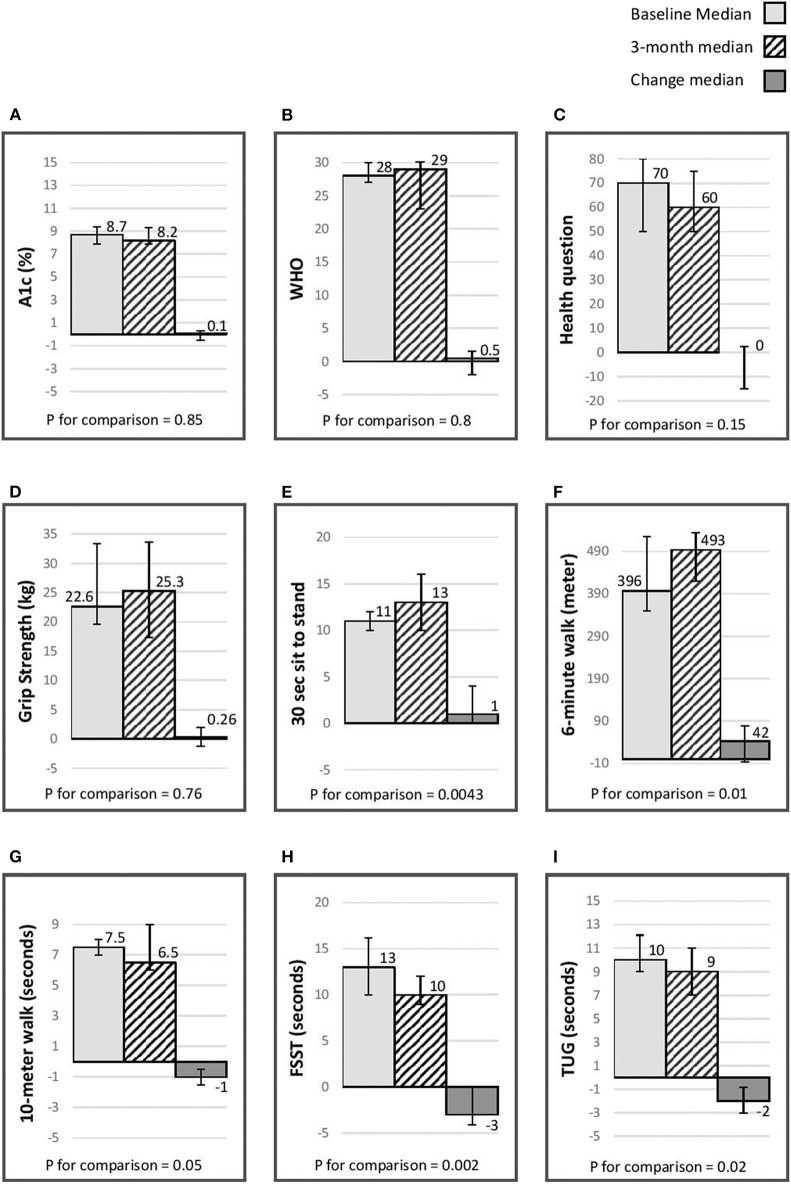
Median (IQR) baseline, 3 month & change (p for comparison) in A1C, quality of life, & physical indices. **(A)** A1C (%); **(B)** WHO-5 well-being questionnaire (26); **(C)** single general self- rated health question (27); **(D)** grip strength(kg) assessed using a Jammer dynamometer (25); **(E)** 30-s chair stand, score is the number of stands in 30 s (24); **(F)** 6-min walk distance (meters) (14–16); **(G)** 10-meter walk (seconds) (17, 18); **(H)** Four Step Square Test (FSST) (seconds) (19); **(I)** timed Up & Go (TUG) (seconds) (20–23).

Average overall satisfaction with the program was 9.5, and score for the question “will the program have an impact on your long-term health” was 9.5. Figure 2 presents the median baseline, 3 months, and the change experienced during the intensive phase in A1C, physical indices and quality of life. There was an improvement in quality of life and a reduction in A1C; however, these changes did not reach statistical significance. There was nevertheless a statistically significant improvement in physical indices, including aerobic capacity (6-min walk), balance (FSST), lower limb strength (30 s sit to stand) and indices assessing the risk of fall (10-meter walk, TUG).

### Difference in Outcomes at the End of Study (12-Months)

Table 3 presents the difference in A1C, physical indices, and quality of life between baseline, 3 months, and at the end of follow-up. There was a 0.7% reduction in A1C from baseline to end of study (*p* < 0.05). There was no change in quality of life. There was a statistically significant reduction in grip strength by the end of the study. For the FSST (dynamic balance) and 30-s chair (lower limb strength) there was an improvement after 3 months and some of the improvement experienced during this interval persisted to end of the study. For the 6-min walk (aerobic capacity) stand there was an improvement after 3 months and at the end of the study. For the TUG (assess risk for falls), there was a statistically significant improvement after 3 months that did not persist to the end of study assessment.

**Table 3:** Change in A1C, physical indices, and quality of life after 3 & 12 months.

**Variable**	**Difference 0–3 M**	**Difference 3–12 M**	**Difference 0–12 M**
A1C	−0.16 (0.21)	−0.56 (0.23)^*^	−0.72 (0.23)^*^
WHO	0.24 (1.23)	−0.16 (1.31)	0.08 (1.30)
Health question	−5.32 (3.50)	4.24 (3.80)	−1.08 (3.78)
Grip Strength (kg)	0.08 (0.65)	−4.19(0.70)^*^	−4.11(0.70)^*^
30-s chair stand	2.27 (0.75)^*^	−0.22 (0.83)	2.10 (0.83)^*^
6-min walk (meters)	39.13 (12.73)^*^	23.70 (13.97)	62.84 (13.94)^*^
10-meter walk (seconds)	−0.40 (0.39)	0.19 (0.43)	−0.21 (0.43)
FSST (seconds)	−2.30 (0.49)^*^	0.67 (0.53)	−1.62 (0.53)^*^
TUG (seconds)	−1.40 (0.55)^*^	1.91 (0.60)^*^	0.52 (0.60)

* *p < 0.05; scores presented are follow-up minus baseline- mean (SE); WHO-5 well-being questionnaire (26); the single general self- rated health question (27); grip strength was assessed using a Jammer dynamometer (25); Four Step Square Test (FSST) (19); 6-min walk (14–16); timed Up & Go (TUG) (20–23); 10-meter walk (17, 18); 30-s chair stand score in number of stands in 30 s (24)*.

## Discussion

A complex intervention conducted in older people with diabetes with sub-optimal glucose control and below-normal cognitive function was feasible and after 12 months demonstrated an improvement in A1C. After 3 months there was an improvement in physical indices related to aerobic capacity, strength and balance and a reduction in the risk for falls, possibly mediated through an improvement in self-care capacity. These improvements were generally diminished or did not persist to the end of study period.

Previous studies in people with and without diabetes have demonstrated the efficacy of a MDT intervention. Thus, the FINGER trial a 2 year multi-domain group intervention of diet, exercise, cognitive training and vascular risk monitoring conducted in people at high risk for cognitive decline, demonstrated that individuals randomized to the intervention arm experienced less cognitive decline then those randomized to the standard care arm and included ~162 (13%) older people with diabetes (35). The short follow-up duration of our study precluded the ability to detect a difference in cognitive decline rates, however a long-term follow with cognitive assessment is planned. Physical exercise programs including resistance and endurance activity tailored to the physical profile of the individual have been shown to reduce the incidence of disability in older people with diabetes. The LIFE trial demonstrated that a structured moderate intensity physical activity program among community dwelling individuals 70–89 years of age who were at high risk for mobility disability reduced the risk for major disability the effect observed in a sub-group of ~450 individuals with diabetes, albeit not significant, was even more pronounced (36). These results are consistent with the results of our study in which a significant improvement was noted in physical indices related to aerobic capacity, strength, balance and a reduction in the risk for falls.

This study has several limitations including its small size, the lack of a control group and the relatively short follow-up, However, it does provide data regarding the feasibility and the possible efficacy of such an intervention including its effects on glucose control and physical indices cardinal to the aging person with diabetes such as aerobic capacity, strength and balance measures. To the best of our knowledge, this study is the first to include cognitive training and rehabilitation as part of a multi-disciplinary approach aimed at improving adherence to self-care management in an older diabetic cohort. This approach enables the older individual to learn compensatory cognitive strategies and adjust the self-care management plan to the cognitive challenges they are facing, while simultaneously utilizing in an optimal way existing cognitive assets.

To conclude this small feasibility study provides preliminary data that supports the efficacy of the complex intervention described. In light of the change in physical indices that did not persist into the consolidation phase, it seems that this population would need an on-going “intensive phase” intervention. On a public health level, given the high prevalence of diabetes among older adults and the fact that this population is at a high risk for disability, even a small improvement in physical indices will have high impact as it may reduce the rates of disability in this high-risk population. Further studies are needed in order to assess how this type of intervention may be applied to a larger population.

## Data Availability Statement

The datasets generated for this study are available on request to the corresponding author.

## Ethics Statement

The studies involving human participants were reviewed and approved by Sheba Medical Center IRB. The patients/participants provided their written informed consent to participate in this study.

## Author's Note

An abstract of the current work was presented as a poster at the annual meeting of the Israel Endocrine Society, 2019.

## Author Contributions

MA, OG, TC-Y, RN, HM, NG, SR, and TY contributed to acquisition and interpretation of data. TC-Y and RN contributed to acquisition, interpretation, analysis of data, and drafting this manuscript. OK contributed to the drafting, revising, and formatting of the manuscript. TC-Y also made substantial contribution to conception and design of the research described. MA, NG, OG, TY, RN, OK, SR, and TC-Y have read and approved the final manuscript.

## Conflict of Interest

The authors declare that the research was conducted in the absence of any commercial or financial relationships that could be construed as a potential conflict of interest.
